# Phylogenetic Analyses and Plastome Comparison to Confirm the Taxonomic Position of *Ligusticum multivittatum* (Apiaceae, Apioideae)

**DOI:** 10.3390/genes16070823

**Published:** 2025-07-14

**Authors:** Changkun Liu, Boni Song, Feng Yong, Chengdong Xu, Quanying Dong, Xiaoyi Wang, Chao Sun, Zhenji Wang

**Affiliations:** 1 College of Resources Environment and Chemistry, Chuxiong Normal University, Chuxiong 675000, China chtown@cxtc.edu.cn (C.X.); dongquanying@cxtc.edu.cn (Q.D.); wangxiaoyi@cxtc.edu.cn (X.W.); sunchao@cxtc.edu.cn (C.S.); 2 Key Laboratory of Bio-Resources and Eco-Environment of Ministry of Education, College of Life Sciences, Sichuan University, Chengdu 610065, China; songboni@stu.scu.edu.cn; 3 School of Life Science and Engineering, Lanzhou University of Technology, Lanzhou 730050, China; lut886@163.com

**Keywords:** *Ligusticum*, *Ligusticum multivittatum*, phylogeny, plastome, taxonomy

## Abstract

Background: *Ligusticum* L. plants exhibit significant morphological variation in leaves, flowers, bracteoles and mericarps, thus the classifications of members for the genus have always been controversial. Among them, the taxonomic problem of *Ligusticum multivittatum* Franch. is the most prominent, which has not been sufficiently resolved so far. Methods: to clarify the taxonomic position of *Ligusticum multivittatum*, we performed phylogenetic analyses based on plastome data and ITS sequences. Meanwhile, we conducted comprehensively comparative plastome analyses between *Ligusticum multivittatum* and fifteen *Ligusticopsis* species. Results: Both analyses robustly supported that *Ligusticum multivittatum* nested in genus *Ligusticopsis* Leute and formed a clade with fifteen *Ligusticopsis* species, belonged to the Selineae tribe, which was distant from the type species of *Ligusticum* (*Ligusticum scoticum*), located in the *Acronema* clade.The comparative results showed that sixteen plastomes were highly similar and conservative in genome structure, size, gene content and arrangement, codon bias, SSRs and SC/IR. These findings imply that *Ligusticum multivittatum* is a member of *Ligusticopsis*, which was further verified by their shared morphological characters: stem base clothed in fibrous remnant sheaths, white petals, pinnate bracteoles, dorsally compressed mericarps with slightly prominent dorsal ribs, winged lateral ribs and numerous vittae in the commissure and in each furrow. Therefore, combining with the evidences of phylogenetic analyses, plastome comparison and morphological features, we affirmed that *Ligusticum multivittatum* indeed belonged to *Ligusticopsis* and transformed it into *Ligusticopsis* conducted by Pimenov was reasonable. Conclusions: Our study not only confirms the classification of *Ligusticum multivittatum* by integrating evidences, but also provides a reference for resolving taxonomy of contentious taxa.

## 1. Introduction

*Ligusticum* L., belonging to the family Apiaceae, was established by Linnaeus in 1753 [1] and Koch designated *Ligusticum scoticum* L. as the type species in 1824 [2]. The genus is one of the largest genera of Apiaceae and contains about 60 species worldwide, with 40 species (35 endemics) in China [3,4,5,6]. Due to the significant morphological variation in leaves, flowers, bracteoles, mericarps of *Ligusticum* plants, species within this genus frequently were transferred between closely related genera. For example, *Ligusticum involucratum* Franch., *Ligusticum hispidum* (Franch.) H. Wolff ex Hand.-Mazz. and *Ligusticum daucoides* (Franch.) Franch. were regarded as independent species of *Ligusticum* since its publication [3,6], but Pimenov incorporated them into the genus *Ligusticopsis* established by Leute [7,8]. Fortunately, Li et al. [9] and Ren et al. [10] recently confirmed taxonomic position of these controversial species of *Ligusticum* based on molecular and morphological evidences.

This study focused on *Ligusticum multivittatum*, which has serious classification problems, and is endemic to China, distributed in Yunnan and Sichuan provinces, growing in areas with an altitude of 3000 to 4100 m, mostly in bamboo forests and grasslands [6]. This species has been treated as an independent *Ligusticum* species since its publication and accepted by some scholars [3,4]. Subsequently, Pimenov transformed the species into *Ligusticopsis* only based on the morphological observation of the specimen [8]. However, Weng et al. still regarded *Ligusticum multivittatum* as a member of *Ligusticum* in recent study [11]. Thus, it can be seen that *Ligusticum multivittatum* has undergone changes between the genera *Ligusticum* and *Ligusticopsis,* which severely hinders the exploration and conservation of germplasm resources for traditional medicinal herbs in *Ligusticum*. The systematic position of this species urgently needs clarification.

Due to the advantage of lacking recombination, having low nucleotide substitution rate and possessing highly variable characteristics, plastome has the potential to improve the support of phylogenetic trees and also provide valuable information for resolving complex relationships in plants [12,13,14,15]. Comparative analysis of plastome can reveal the structure and characteristics of plastome that broaden our understanding of adaptive evolution of plant lineages and screen out highly mutation hotspots regions for species identification [16]. In recent years, with the advancement of next-generation sequencing technology, obtaining the plastome data has become more accessible [17] and it has been widely and successfully used in phylogenomic studies, especially for those taxonomic position contentious taxa, such as *Seseli* L. [18], *Peucedanum* L. [19], *Elatostema* J. R. Forst. & G. Forst. [20] and *Salix* L. [21].

In this study, we conducted phylogenetic analyses based on plastome data and ITS sequences to reveal the phylogenetic position of *Ligusticum multivittatum*. Then, a comprehensive comparison analyses between *Ligusticum multivittatum* plastome and fifteen *Ligusticopsis* plastomes were performed to characterize the plastomes of *Ligusticum multivittatum* and *Ligusticopsis* plants. Finally, combining with phylogenetic analyses, comparative plastome analyses and morphological characteristics, we clarified the taxonomic placement of *Ligusticum multivittatum*.

## 2. Materials and Methods

### 2.1. Phylogenetic Analysis

To confirm the taxonomic placement of *Ligusticum multivittatum*, we used two datasets to reconstruct the phylogenetic trees. Dataset 1 was 66 plastome sequences and dataset 2 was 69 ITS sequences (Table S1). According to the previous study, *Bupleurum chinense* Franch. and *B. falcatum* L. were selected as outgroups [22]. The software MAFFT v7.221 [23] was used to align the ITS and plastome sequences and adjusted manually when necessary. Maximum likelihood analysis (ML) and Bayesian inference (BI) were used to perform the phylogenetic analyses. For ML analysis, referring to the RAxML manual, RAxML v8.2.8 [24] with 1000 replicates and GTRGAMMA model was used to reconstruct the phylogenetic tree and estimate the value of bootstrap support (BS) for each node. For BI analysis, MrBayes v3.2.7 [25] was performed to reconstruct the phylogenetic trees and the best-fit substitution model (GTR+I+G) for both datasets were determined by Modeltest v3.7 [26]. Two independent Markov chain Monte Carlo (MCMC) algorithms were run for 1,000,000 generations, sampling every 100 generations. When the average standard deviation of the splitting frequency dropped below 0.01, the MCMC running finished. Then, we discarded the initial 25% of trees and used the reminders to yield the consensus tree and estimate posterior probabilities (PP). Finally, the software FigTree v1.4.2 [27] and the online tool iTOL (https://itol.embl.de/itol.cgi, accessed on 2 May 2025) [28] were used to edit and visualize the phylogenetic trees.

### 2.2. Plastome Comparison Analyses

We downloaded sixteen plastomes (*Ligusticum multivittatum* plastome and fifteen *Ligusticopsis* plastomes) from NCBI databases (Table S1) and annotated them using Geneious R11 (Biomatters Ltd., Auckland, New Zealand) [29]. For plastome comparison analyses, we first compared the IR/SC boundaries of these sixteen plastome sequences using Geneious R11 (Biomatters Ltd., Auckland, New Zealand) [29] and visualized it after manually adjusted. Then, we detected the possible gene rearrangements among these sixteen sequences using the whole genome alignment tool Mauve v1.1.3 plugin [30] in Geneious R11 (Biomatters Ltd., Auckland, New Zealand) [29], setting other parameters as the default values. Finally, we investigated the degree of variation sequences of these sixteen sequences using the LAGAN model implemented in mVISTA tool [31] with *Ligusticopsis brachyloba* (Franch.) Leute as the reference.

### 2.3. Simple Sequence Repeats and Codon Bias Analyses

We used MISA Perl script [32] to calculate the simple sequence repeats (SSRs) of each plastome sequence. And the minimum number of repeat units was set to 10, 5, 4, 3, 3 and 3, for mono-, di-, tri-, tetra-, penta- and hexanucleotides, respectively.

For codon bias analysis, we extracted the coding sequence (CDSs) from each plastome sequence using software Phylosuite v1.2.2 [33]. To avoid sampling bias and shorter sequences may bias the codon usage estimation, we deleted all overlapping sequences, removed the sequences shorter than 300 bp and finally screened out 53 CDSs to performed codon bias analysis. The codon bias for each plastome sequence was conducted by CodonW v1.4.2 [34] and the result was visualized by TBtools v2.309 [35].

### 2.4. Identification of Divergence Hotspots

To identify divergence hotspot, the protein-coding regions, the non-coding regions, and the intergenic regions were extracted and aligned using Phylosuite v1.2.2 [33]. Then, DnaSP v5.0 [36] was used to calculate the nucleotide diversity (Pi) of aligned sequences with more than 200 bp in length.

## 3. Results

### 3.1. Phylogenetic Analyses

**Figure 1:**
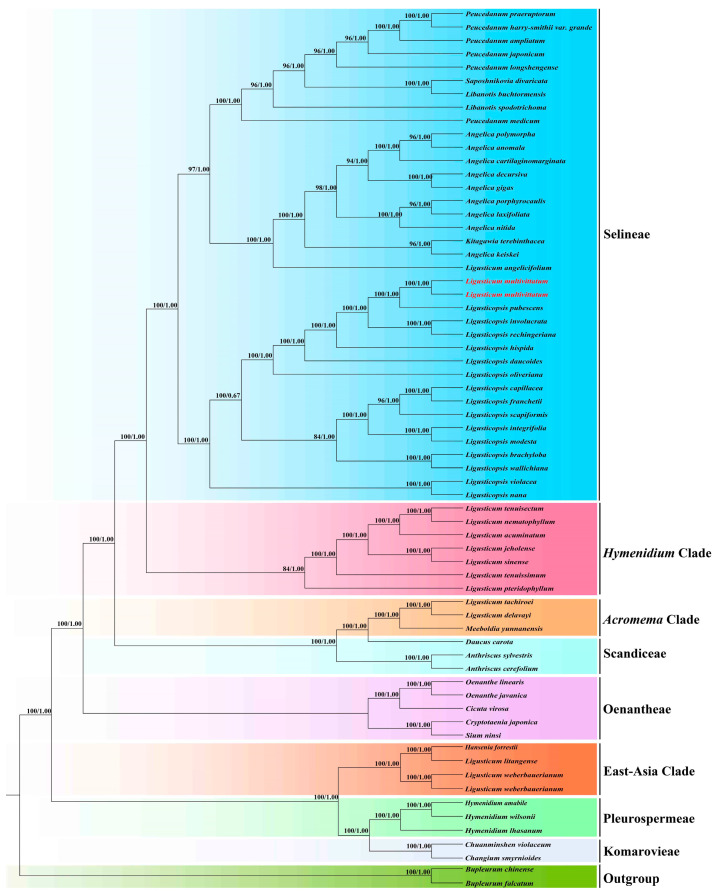
Phylogenetic tree inferred from plastome data. Numbers represent maximum likelihood bootstrap values (BS) and Bayesian posterior probabilities (PP).

**Figure 2:**
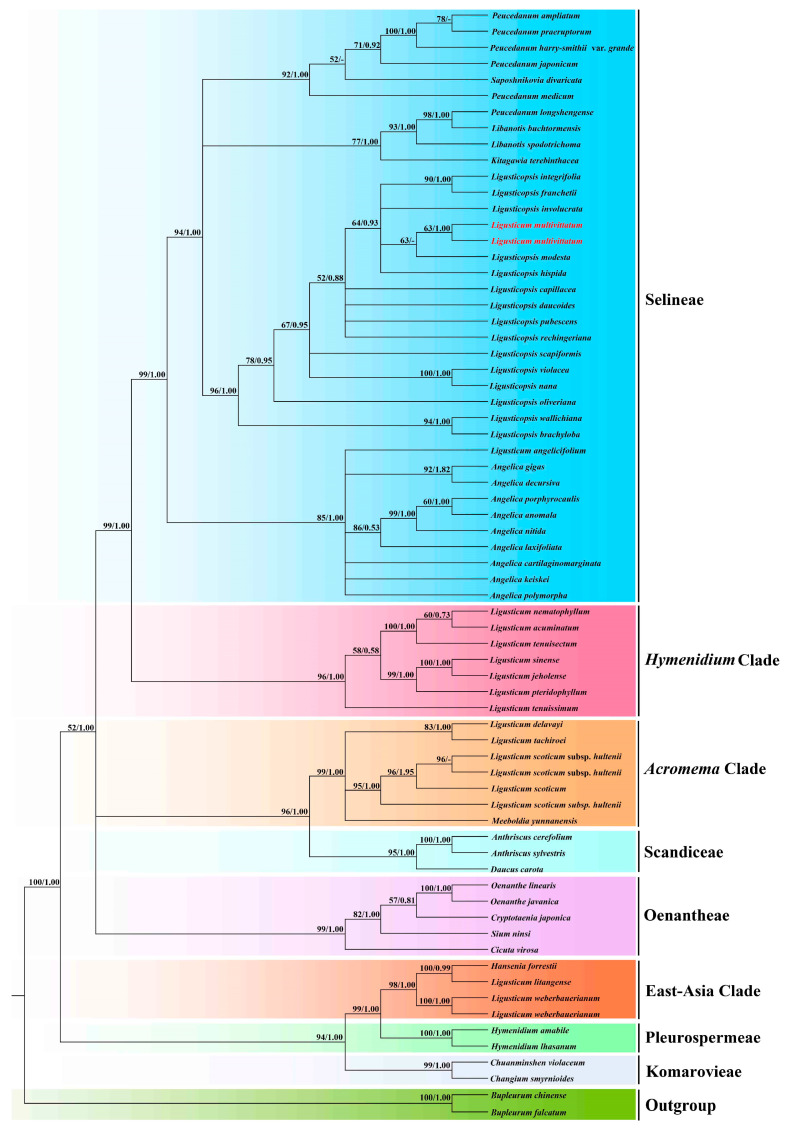
Phylogenetic tree inferred from ITS sequences. Numbers represent maximum likelihood bootstrap values (BS) and Bayesian posterior probabilities (PP). - represents the values < 50/0.50.

In the ITS-based and plastome-based phylogenetic tree, the analyses of ML and BI yielded identical tree topologies, respectively (Figure 1 and Figure 2). Although the tree topologies of the plastome data and ITS sequences were incongruent, both strongly showed that *Ligusticum multivittatum* clustered with fifteen *Ligusticopsis* species and formed a clade with high supports (plastome: BS = 100, PP = 1.00; ITS:BS = 96, PP = 1.00) within the Selineae tribe, which was obviously distant from other *Ligusticum* taxa. In the ITS-based phylogenetic tree, *Ligusticum multivittatum* was sister to *Ligusticopsis modesta* (BS = 63, PP < 0.50), located in the Selineae tribe. And this clade was distant from the *Acronem*a clade that *Ligusticum scoticum* L. (the type species of *Ligusticum* L.) was located in (Figure 2). While in plastome-based phylogenetic tree, *Ligusticum multivittatum* was sister to *Ligusticopsis pubescens* with robust supports (BS = 100, PP = 1.00) (Figure 1). In addition, *Ligusticum* taxa involved in this study scattered in four clades (Selineae tribe, *Hymenidium* clade, *Acronema* clade, East-Asia clade).

### 3.2. Characteristics of Plastome

The study comprehensively compared *Ligusticum multivittatum* plastome with fifteen *Ligusticopsis* plastomes. The results showed that all of them had a typical quadripartite structure (LSC: 91,480–92,305 bp; SSC: 16,335–17,691 bp; IRs: 19,056–20,022 bp). The genome length ranged from 146,900 bp (*Ligusticopsis nana*) to 148,633 bp (*Ligusticopsis brachyloba*). The total GC content was 37.4–37.5%, and the GC content in the IR region was the highest (43.6–44.2%) compared to the LSC (35.9–36.0%) and SSC regions (30.8–31.0%). In addition, the gene number was highly consistent among sixteen plastomes (79 protein-coding genes, 30 tRNA genes, and four rRNA genes) (Table 1). Therefore, the plastome structure, number, length and GC content were largely similar across the sixteen species.

### 3.3. Simple Sequence Repeats and Codon Bias

**Figure 3:**
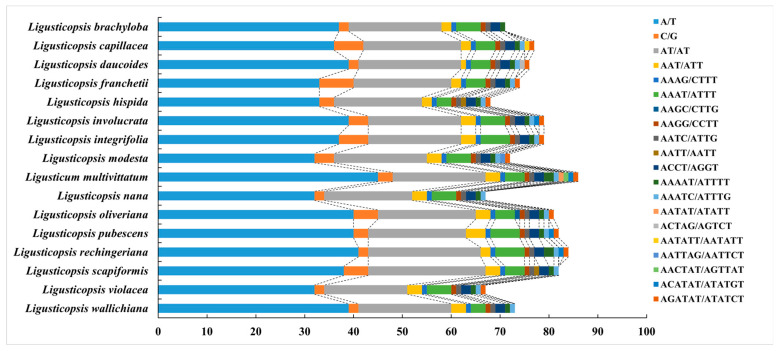
Analysis of simple sequence repeats (SSRs) in sixteen plastomes.

In this study, we investigated the SSRs of sixteen plastome and the results showed that the number of SSRs was similar among them (Figure 3, Table S2). The total number of SSRs varied from 67 (*Ligusticopsis violacea*, *Ligusticopsis nana*) to 86 (*Ligusticum multivittatum*) (Table S2). Six repeat types were detected in sixteen plastomes, in which mono-repeat was the most (36–48) while hexa-repeat was the least (0–3). Notably, hexa-repeat was absent in four plastome (*Ligusticopsis brachyloba*, *Ligusticopsis nana*, *Ligusticopsis scapiformis*, *Ligusticopsis wallichiana*) (Figure 3).

**Figure 4:**
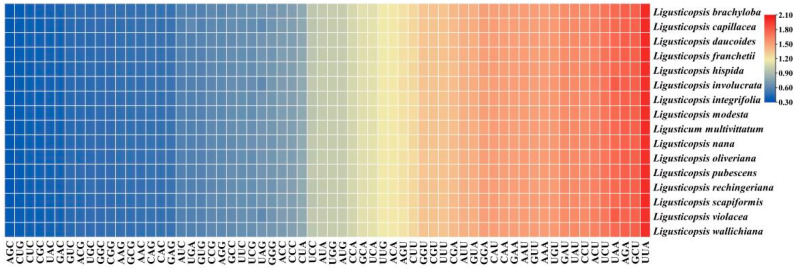
RSCU values of sixteen plastomes. Color key: Red represents higher RSCU values while blue represents lower RSCU values.

In addition, 53 CDSs with length longer than 300 bp were used to conduct the codon bias analysis and the results revealed that sixteen species had highly consistent codon usage patterns (Figure 4, Table S3). In detail, these sequences encoded 21,058–21,159 codons and *Ligusticum multivittatum* had the most codons while *Ligusticopsis involucrata* had the least codons. Among these codons, Leu was encoded by the most codons (2235–2245) while Cys was encoded by the least codons (211–218) (Table S3). Moreover, the relative synonymous codon usage (RSCU) value among these sixteen plastomes was also similar and it varied from 0.34 to 2.01. Among them, 30 codons had RSCU value greater than 1.00 and they were regarded as preference codons, with all of them ended with A/U. The RSCU value of two codons (UGG and AUG) was equal to 1.00 and had no preference. The remaining 32 codons had RSCU value less than 1.00 (Figure 4).

### 3.4. Plastome Comparison

**Figure 5:**
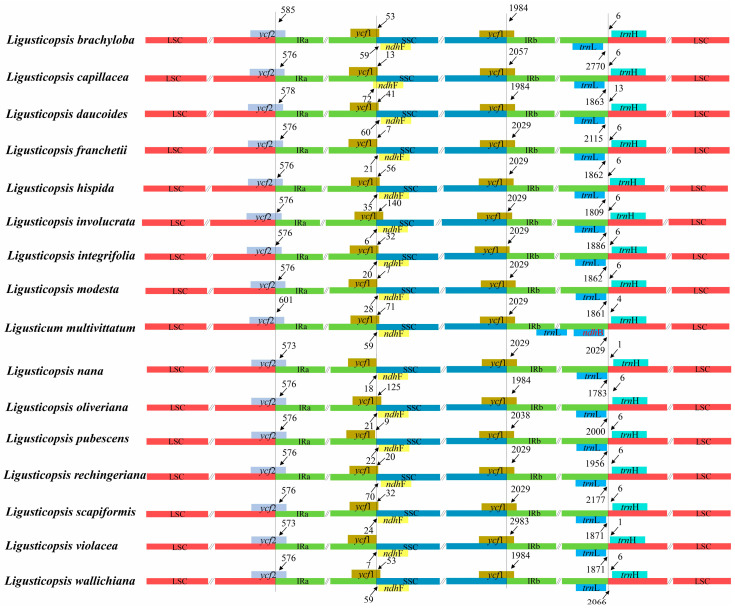
Comparative analyses of LSC, SSC and IR region boundaries for sixteen plastomes.

The analysis of the borders of LSC/IRs and SSC/IRs showed that LSC/IRa, IRa/SSC, SSC/IRb had similar IR boundaries and the adjacent genes were also identical (Figure 5). In detail, *ycf*2 gene crossed the LSC/IRa junction and expanded 573–601 bp into IRa region. At the IRa/SSC boundary, except for the *ndh*F gene of *Ligusticopsis capillacea* expanding to the IR region with length of 72 bp, the *ndh*F gene of other species were far away from the IRa/SSC boundary, with length of 6–70 bp. The *ycf*1 gene, crossing the SSC/IRb junction, expanded 1984–2983 bp into the IRb region. IRb/LSC boundary was located between *trn*L and *trn*H genes in fifteen *Ligusticopsis* plastomes, with the *trn*L gene were 1783–2770 bp away from LSC region and *trn*H gene had 1–13 bp away from IRb regions, while in *Ligusticum multivittatum* plastome, the IRb/LSC border was located between *ndh*B and *trn*H genes and *ndh*B was far away from LSC region with 2029 bp (Figure 5).

**Figure 6:**
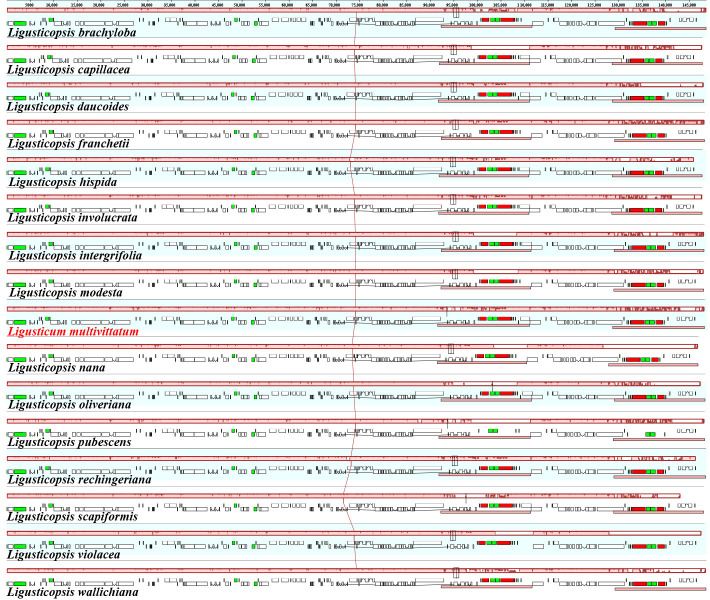
Mauve alignment of sixteen plastomes. Local collinear blocks within each alignment are represented by blocks of the same color connected with lines.

Moreover, mauve alignment results revealed that no rearrangement or loss were detected and these sixteen plastomes were highly conserved (Figure 6).

**Figure 7:**
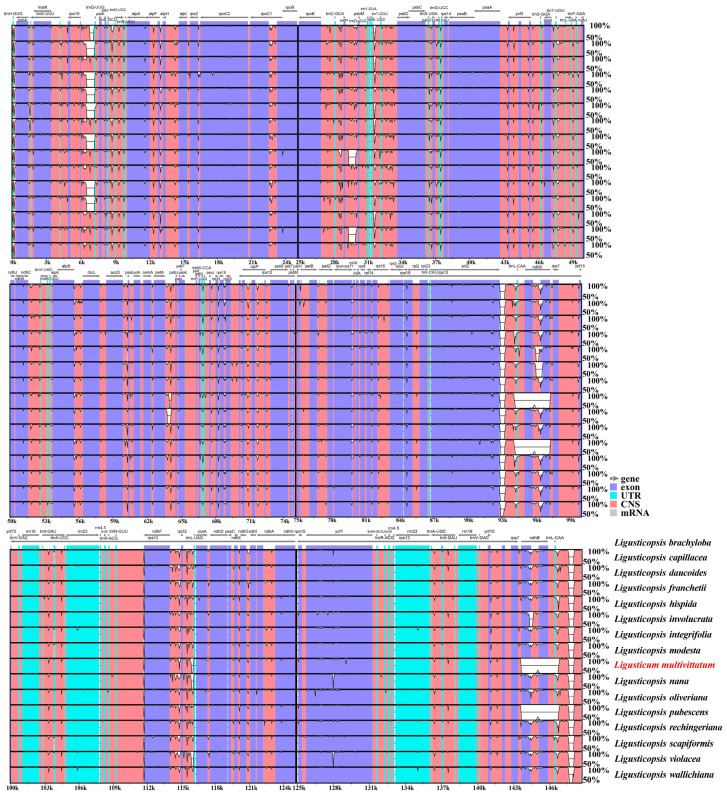
Sequence identity plots for sixteen plastomes using mVISTA. *y*-axis corresponds to percentage identity (50–100%), while *x*-axis shows position of each region within the locus. Arrows indicate transcription of annotated genes in reference genome.

Furthermore, mVISTA results showed that these sixteen plastomes sequences were highly similar and no significant variations were found (Figure 7).

### 3.5. Nucleotide Diversity (Pi) Analysis

**Figure 8:**
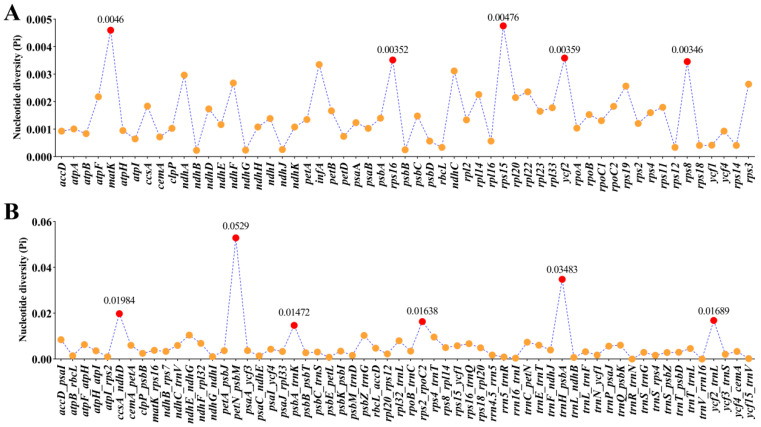
Comparative analysis of nucleotide diversity (Pi) values among sixteen plastomes. (**A**): protein coding regions; (**B**): non-coding and intron regions.

Although these sixteen plastomes were highly conserved, this study also identified eleven high mutation regions based on nucleotide diversity analysis that can acted as potential DNA barcodes for species identification in *Ligusticopsis* genus (Figure 8). The eleven high mutation regions included five protein-coding regions (*mat*K, *rps*16, *rps*15, *ycf*2, *rps*8) that had Pi values of 0.0046, 0.00352, 0.00476, 0.00359 and 0.00346 and six non-coding regions (*ccs*A-*ndh*D, *pet*N-*psb*M, *psb*A-*trn*K, *rps*2-*rpoC*2, *trn*H-*psb*A, *ycf* 2-*trn*L) that showed Pi values of 0.01984, 0.0529, 0.01472, 0.01638, 0.03483 and 0.01689. In addition, Pi values in protein-coding regions (Figure 8A) were lower than in non-coding regions (Figure 8B).

## 4. Discussion

### 4.1. Plastome Comparative Analyses

Comparative plastome analyses can provide useful information for understanding the molecular evolution patterns of plants [37,38]. In this study, sixteen plastomes had similar quadripartite structure (one LSC region, one SSC region and two IRs region) that was also detected in other genera of Apiaceae, such as in *Acronema* Edgew. [22], *Sanicula* L. [19,39], *Libanotis* Haller ex Zinn [40] and so on. In addition, although IR contraction or expansion, gene loss and arrangement are common evolutionary events that frequently occurs in other genera of Apiaceae [41,42], the analyses of IR borders, simple sequence repeats, codon bias, whole genome alignment and sequences variation of these sixteen plastomes illustrated that they were all highly similar. The finding of these highly conserved and similar plastomes implied that *Ligusticum multivittatum* may be a member of genus *Ligusticopsis.*

Although these sixteen plastomes were highly similar and conserved, we still selected eleven high mutation regions (*mat*K, *rps*16, *rps*15, *ycf*2, *rps*8, *ccs*A-*ndh*D, *pet*N-*psb*M, *psb*A-*trn*K, *rps*2-*rpo*C2, *trn*H-*psb*A, *ycf*2-*trn*L) that could be acted as the candidate DNA barcodes for species identification in *Ligusticopsis* genus. Of these, *mat*K and *trn*H-*psb*A are considered as universal DNA barcodes [43], other nine regions can used as the specific DNA barcodes to discriminate *Ligusticopsis* species and can also use for phylogeny in future research.

### 4.2. Phylogenetic Inference and Taxonomic Implication of Ligusticum Multivittatum

A robust phylogenetic topology could provide convincing evidence to clarify taxonomic controversy of species. However, using single or a few fragments always obtain phylogenetic tree with weak supports and low resolutions [44,45,46,47]. Plastomes with highly variable characters and cost-effective sequencing thus were widely used for phylogenetic analysis to generate robust and stable phylogenetic framework [48,49,50,51,52]. In the current study, we performed phylogenetic analysis based on plastome data for *Ligusticum multivittatum*. As expected, compared to previous study by using single fragment [43], our phylogenetic tree based on plastome data exhibited high supports and resolutions. Meanwhile, we also conducted phylogenetic analysis by using ITS sequence to remedy the weakness of uniparental inheritance of plastome data. Integrating plastome-based tree with ITS-based tree, phylogenetic position of *Ligusticum multivittatum* was elucidated. Our results provide a reference to confirm the phylogenetic position of taxonomic controversial species.

Our phylogenetic analyses based on plastome data and ITS sequences robustly supported that *Ligusticum multivittatum* fell into genus *Ligusticopsis* with high supports, belonged to the Selineae tribe. But the type species of *Ligusticum* (*Ligusticum scoticum*) was located in *Acronema* clade in the ITS-based phylogenetic tree that was distant from *Ligusticum multivittatum*. In addition, plastome comparative analyses showed *Ligusticum multivittatum* had highly similar genome structure, size, codon bias, SSRs, gene content and arrangement and SC/IR with the fifteen plastomes of *Ligusticopsis*. Furthermore, the affinity between *Ligusticum multivittatum* and *Ligusticopsis* was supported by the shared morphological features, such as stem base clothed in fibrous remnant sheaths, white petals, pinnate bracteoles, oblong fruit, dorsally compressed mericarps with slightly prominent dorsal ribs, winged lateral ribs and numerous vittae in the commissure and in each furrow [3,6,9]. However, *Ligusticum multivittatum* has its distinctive characteristics: numerous stems, 1–2-branched or unbranched, the terminal lobes of the basal leaves are lanceolate, 1–2 cauline leaves, smaller or absent, and 7–15 rays [3,6], which can easily distinguish it from other *Ligusticopsis* members. Therefore, by integrating the evidences of phylogenetic analyses, plastome comparison and morphological features, we confirmed that *Ligusticum multivittatum* indeed belonged to *Ligusticopsis* and transferring it into *Ligusticopsis* performed by Pimenov [8] was reasonable.

## 5. Conclusions

In this study, we conducted phylogenetic analyses based on two datasets (plastome data and ITS sequences) and comprehensively plastome comparison for *Ligusticum multivittatum* and closely related taxa. Phylogenetic topologies showed that *Ligusticum multivittatum* nested into *Ligusticopsis* genus, which was further justified by the highly similarity of plastome features between *Ligusticum multivittatum* and *Ligusticopsis* members. Furthermore, the affinity among them was also verified by their shared morphological features. Therefore, *Ligusticum multivittatum* is indeed a member of *Ligusticopsis* genus and the taxonomic revision performed by Pimenov was reasonable. Additionally, we selected eleven high mutation regions that could act as the potential DNA barcodes for *Ligusticopsis* species identification. Overall, our results not only confirmed the taxonomy of *Ligusticum multivittatum* to lay the foundations for future species identification, phylogeny and breeding of *Ligusticum*, but also provide a reference for resolving taxonomy of contentious taxa.

## Figures and Tables

**Table 1:** Features of sixteen plastomes.

Taxon	Length (bp)					Number of Genes (Unique)			
	Genome	LSC	SSC	IR	Total	CDS	rRNA	tRNA	GC (%)
*Ligusticopsis brachyloba*	148,633	92,265	17,588	19,390	113	79	4	30	37.40%
*Ligusticopsis capillacea*	147,808	91,907	17,503	19,199	113	79	4	30	37.50%
*Ligusticopsis daucoides*	148,078	91,666	17,582	19,415	113	79	4	30	37.40%
*Ligusticopsis franchetii*	148,281	92,298	17,691	19,146	113	79	4	30	37.50%
*Ligusticopsis hispida*	147,797	91,846	17,627	19,162	113	79	4	30	37.40%
*Ligusticopsis involucrata*	147,752	91,782	17,560	19,205	113	79	4	30	37.40%
*Ligusticopsis intergrifolia*	148,196	92,305	17,575	19,158	113	79	4	30	37.50%
*Ligusticopsis modesta*	148,133	92,247	17,568	19,159	113	79	4	30	37.50%
*Ligusticopsis nana*	146,900	91,480	17,308	19,056	113	79	4	30	37.50%
*Ligusticopsis oliveriana*	148,175	92,273	17,534	19,184	113	79	4	30	37.50%
*Ligusticopsis pubescens*	148,260	91,819	17,619	19,411	113	79	4	30	37.4%
*Ligusticopsis rechingeriana*	148,525	91,813	17,654	19,529	113	79	4	30	37.30%
*Ligusticopsis scapiformis*	148,107	92,214	17,581	19,156	113	79	4	30	37.50%
*Ligusticopsis violacea*	148,190	91,811	16,335	20,022	113	79	4	30	37.50%
*Ligusticopsis wallichiana*	148,594	92,281	17,567	19,373	113	79	4	30	37.40%
*Ligusticum multivittatum*	148,262	91,571	17,631	19,530	113	79	4	30	37.40%

## Data Availability

The data used in this study were downloaded from NCBI dataset.

## Supplementary Materials

The following supporting information can be downloaded at: https://www.mdpi.com/article/10.3390/genes16070823/s1, Table S1. Sequences of plastomes and ITS obtained from NCBI; Table S2. Numbers of SSR motifs identified in the sixteen plastomes; Table S3. Codon usage and relative synonymous codon usage (RSCU) values of protein-coding genes for the sixteen plastomes.
